# Unusual imaging characteristics of thoracic hydatid disease

**DOI:** 10.1590/0100-3984.2021.0041

**Published:** 2022

**Authors:** Sadullah Şimşek, Cihan Akgül Özmen

**Affiliations:** 1 Department of Radiology, Medical School, Dicle University, Diyarbakır, Turkey.

**Keywords:** Echinococcosis/diagnostic imaging, Echinococcosis, pulmonary/diagnostic imaging, Thoracic diseases/diagnostic imaging, Tomography, X-ray computed, Magnetic resonance imaging, Equinococose/diagnóstico por imagem, Equinococose pulmonar/diagnóstico por imagem, Doenças torácicas/diagnóstico por imagem, Tomografia computadorizada, Ressonância magnética

## Abstract

Cystic echinococcosis (hydatid disease) is a zoonotic parasitic disease, caused by ingestion of *Echinococcus granulosus* eggs, that can result in cyst formation anywhere on the body. Hydatid disease is frequently seen in regions where there is human-animal contact and poor socioeconomic development. The prevalence of the disease ranges from 0 to 79 cases/100,000 population. Hydatid cysts are typically found in the liver and lungs, being less common in other parts of the body. Computed tomography or magnetic resonance imaging is often used in order to clarify the sites affected by a hydatid cyst, such as the cranial and thoracic regions, which also facilitates the surgical evaluation and minimizes complications. Although rare, hydatid cysts in atypical locations can provoke unusual complications, with unpredictable findings and symptoms. This essay discusses the radiological aspects of rare thoracic hydatid cysts.

## Abstract (pt)

A equinococose cística (doença hidática) é uma doença parasitária zoonótica causada pela ingestão de ovos de *Echinococcus granulosus*, que pode resultar na formação de cistos em qualquer parte do corpo. A doença hidática é frequentemente vista em regiões onde há contato humano-animal e baixo desenvolvimento socioeconômico. A prevalência da doença varia de 0 a 79 casos/100.000 habitantes. Os cistos hidáticos são normalmente encontrados no fígado e nos pulmões, sendo menos comuns em outras partes do corpo. A tomografia computadorizada ou a ressonância magnética são frequentemente utilizadas para indicar os locais afetados por um cisto hidático, como as regiões craniana e torácica, o que também facilita a avaliação cirúrgica e minimiza complicações. Embora raros, os cistos hidáticos em locais atípicos podem provocar complicações incomuns, com achados e sintomas imprevisíveis. Este ensaio discute os aspectos radiológicos dos raros cistos hidáticos no tórax.

## INTRODUCTION

Cystic echinococcosis (hydatid disease) is a zoonotic parasitic disease, caused by ingestion of *Echinococcus granulosus* eggs, that can result in cyst formation anywhere on the body. Hydatid cysts are more common in areas where animal husbandry is common and hygiene is poor. The Mediterranean basin is a major endemic area for hydatid disease. The prevalence of the disease ranges from 0 to 79 cases/100,000 population^(1)^. Hydatid cysts are typically found in the liver and lungs, being less common in other parts of the body.

Although hydatid disease is a benign condition, the location and size of a hydatid cyst can change the clinical presentation considerably and result in serious complications, high morbidity, and even death. A study conducted by the World Health Organization in 2010 showed that human echinococcosis can be a serious disease with a reported mortality rate of 4%, most deaths being due to anaphylactic shock caused by cyst rupture. However, that rate is lower in regions with high levels of regional and socioeconomic development^(2)^.

The radiological examination plays an important role in the diagnosis of hydatid disease. Chest X-ray, ultrasound, computed tomography (CT), and magnetic resonance imaging (MRI) are used to diagnose, determine the location and screen of hydatid cysts. Ultrasound is a widely used, well-accepted, noninvasive, affordable, and reproducible imaging method for diagnosing the disease. However, CT or MRI is frequently used in order to clarify the sites affected by a hydatid cyst, such as the cranial or thoracic regions, which also facilitates the surgical evaluation and minimizes complications.

**Figure 1:**
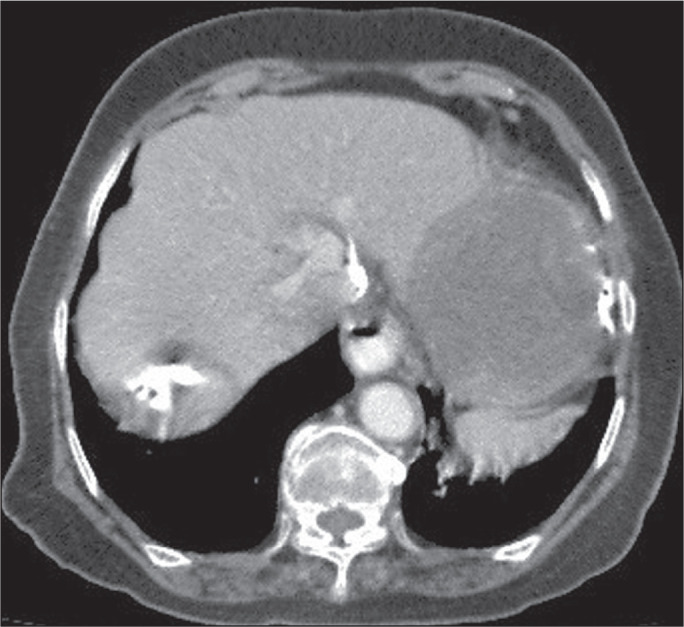
Chest CT scan showing features consistent with hydatid cysts in the left lobe of the liver, with irregular calcification at the periphery.

**Figure 2:**
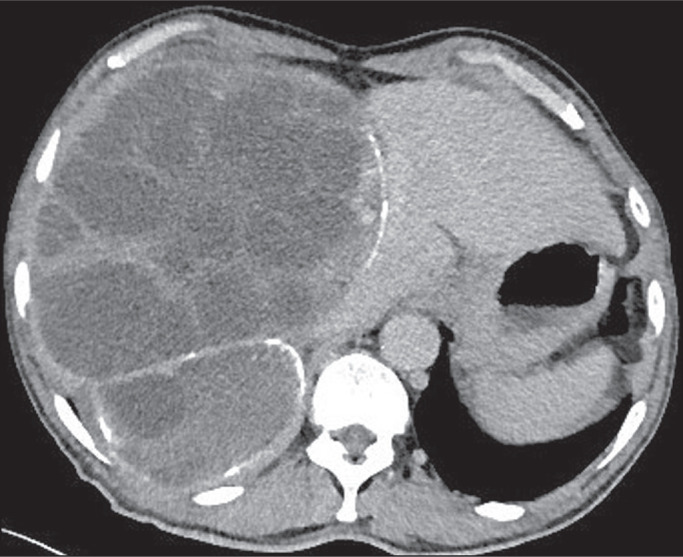
Chest CT scan showing a hydatid cyst, containing multiple daughter cysts, in the right lobe of the liver.

The imaging findings of hydatid cysts are variable. On CT, an uncomplicated hydatid cyst presents as a well-defined homogeneous lesion with low density and smooth walls of variable thickness. After rupture or partial rupture, a hydatid cyst presents the crescent sign, inverse crescent sign, air bubble sign, onion peel (cumbo) sign, spin (whirl) sign, camalote (water-lily) sign, rising sun sign, ball of yarn (mass within a cavity) sign, or empty (dry) cyst sign. Calcifications (Figure 1), daughter cysts (Figure 2), and detached membranes are rare findings that are specific for hydatid cysts. In the presence of these findings, hydatid cysts are classified, according to their radiological appearance, as follows^(3)^: type 1, comprising simple homogeneous cysts; type 2, comprising daughter cysts and cysts with hydatid sand (matrix); type 3, comprising calcified cysts; and type 4, comprising complicated and ruptured cysts.

Hydatid cysts can be located anywhere in the body^(4)^: 50-70% occur in the liver; 11.0-17.0% occur in the lungs; 2.4-5.3% occur in soft tissues; 0.5-3.0% occur in the heart; 1.0-5.0% occur in the pericardium; and 0.5-4.7% occur in the muscles or subcutaneous tissue. The wide range of incidence rates is due to differences among countries and regions. In this pictorial essay, the cases were in individuals of various ages, of different genders, and with various contact patterns. We discuss our radiological experience with rare cases of hydatid disease in which there is thoracic involvement.

## PULMONARY ARTERY

**Figure 3:**
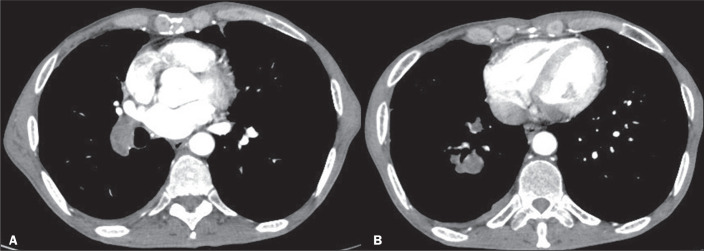
A 57-year-old male patient with a hydatid cyst resulting from contact with an animal. Pulmonary CT angiography showing the hydatid cyst extending from the distal part of the right main pulmonary artery (A) toward the segmental arteries (B).

A hydatid cyst in the pulmonary artery is extremely rare and may cause life-threatening complications^(5)^. The right atrium, right ventricle, and liver have been reported to be embolic sources of hydatid cysts^(6)^. Figure 3 shows a CT scan of a patient with an embolic hydatid cyst completely filling the distal part of the right main pulmonary artery and segmental branches, without involving the heart. The patient presented with a one-month history of dyspnea and hemoptysis. There was a hydatid cyst in the liver, pulmonary CT angiography showed filling defects in the pulmonary arteries, and there was another hydatid cyst that had apparently ruptured in the liver, which was identified as the source of embolism. The results of a test for hydatid cyst-specific immunoglobulin E (IgE) and an enzyme-linked immunosorbent assay were positive.

## INTERCOSTAL SPACE

**Figure 4:**
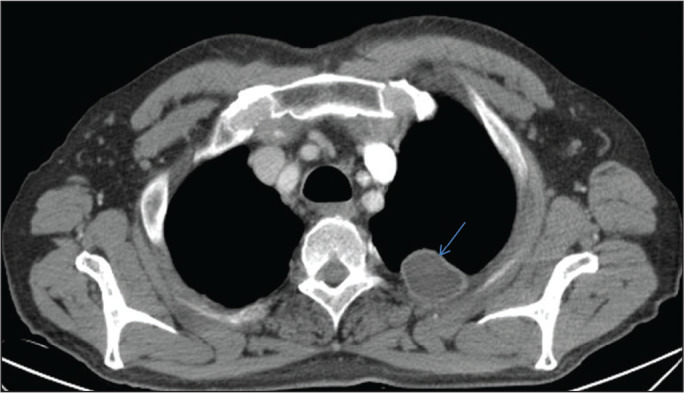
A 65-year-old male patient with a hydatid cyst of unknown origin. Contrast-enhanced chest CT showing a hydatid cyst in the chest wall, occupying the 2nd and 3rd left intercostal spaces (arrow).

Another rare location for a hydatid cyst is in the chest wall. In two large systematic reviews^(7,8)^, evaluating 6,500 and 8,000 cases, respectively, the incidence of chest wall involvement was found to be 0.09% and 3.4%, respectively. The patient had a three-month history of a persistent cough that did not resolve despite medical treatment (Figure 4) and therefore underwent surgical treatment. The diagnosis of hydatid cyst was confirmed on the basis of the pathology examination of the surgical specimen.

## PARAVERTEBRAL AND SPINAL REGIONS

### Paravertebral region

**Figure 5:**
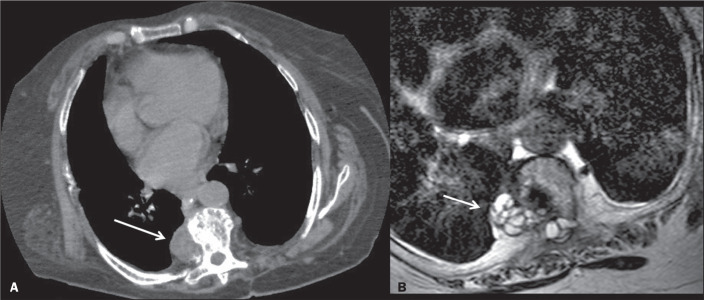
A 45-year-old female patient with a hydatid cyst resulting from contact with an animal. Unenhanced chest CT, acquired because of a trauma, showing a hypodense soft tissue lesion (A), as an incidental finding, in the right paravertebral region, destroying the adjacent (T8) vertebra. An MRI scan of the thoracic-spinal region, showing a multilocular hydatid cyst in the paravertebral space (B), extending toward the neural foramen.

Hydatid cysts are rarely seen in the paravertebral region. Paraspinal and spinal hydatid cysts were first described by Chaussier in 1807^(9)^. Cases of an extraparenchymal hydatid cyst in the thoracic region are difficult to manage. Spinal and paraspinal involvement is also rare, with an incidence of less than 1%^(10)^. In the case presented here (Figure 5), a hydatid cyst was detected incidentally in the right paravertebral location, destroying the adjacent (T8) vertebra. The patient underwent surgery, and the diagnosis of hydatid cyst was confirmed on the basis of the pathology examination of the surgical specimen.

### Paravertebral and spinal region

**Figure 6:**
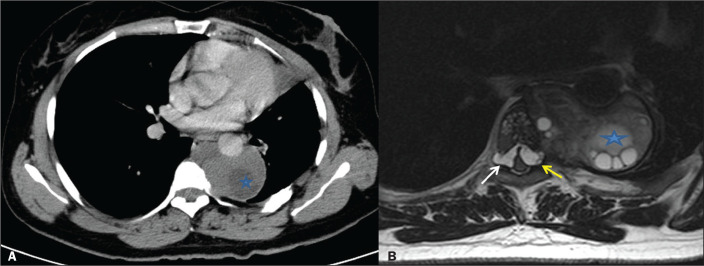
A 38-year-old female patient with a hydatid cyst resulting from contact with an animal. Chest CT (A) showing a hydatid cyst with daughter vesicles (asterisk) in the left paravertebral space, extending to the neural foramen and spinal canal, surrounding the descending aorta and causing destructive changes in the T6 vertebral body. Axial T2-weighted MRI (B), performed for a detailed examination of the entire spinal cord, showing a cystic lesion in the spinal canal, extending to the anterior part of the spinal cord in the left neural foramen (yellow arrow) and to the right neural foramen (white arrow).

Spinal hydatid disease is rare and typically involves the thoracic vertebrae^(11)^. Any part of the spine can be affected. The main presentation/complication is spinal cord compression^(12)^. Patients usually present with back pain and paraparesis. In the case presented here (Figure 6), a patient with a history of hydatid cysts in the liver and lungs presented with complaints of back pain and numbness in the feet. The patient underwent surgery, and the diagnosis of hydatid cyst was confirmed on the basis of the pathology examination of the surgical specimen.

## LUNG

**Figure 7:**
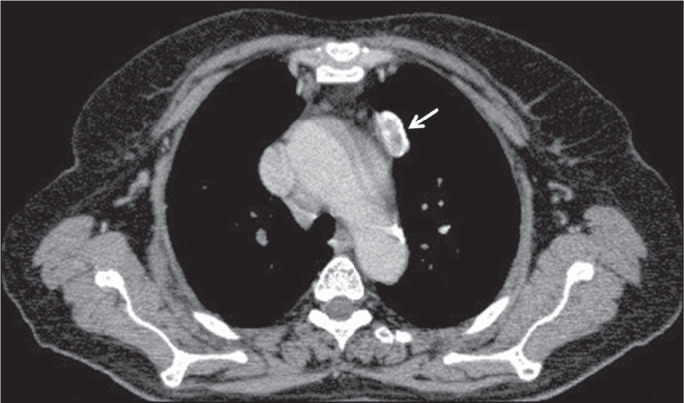
A 71-year-old female with a hydatid cyst of unknown origin. Chest CT showing a peripheral calcified hypodense cyst hydatid (arrow) in the upper lobe of the left lung.

Pulmonary hydatid cysts are detected in 11-17% of all cases hydatid disease. However, calcified pulmonary hydatid cysts are observed in only 0.7% of cases^(13)^. In a patient who presented complaining of a cough, multiple hydatid cysts were identified in the liver and in one lung. In addition, a calcified hydatid cyst was observed in the upper lobe of the left lung (Figure 7).

## HEART/PERICARDIUM

Only 0.5-3.0% of all hydatid cysts occur in the heart^(4)^. The diagnosis of cardiac hydatid disease is based on the combination of clinical suspicion, serological tests, and cardiac imaging. Echocardiography is highly sensitive and specific for the diagnosis of hydatid cysts, and positive serological tests can facilitate the diagnosis^(14,15)^. In the cases evaluated here, the diagnosis of hydatid cyst was made on the basis of cardiac imaging, serological test positivity, and clinical guidance.

### Right atrial involvement

**Figure 8:**
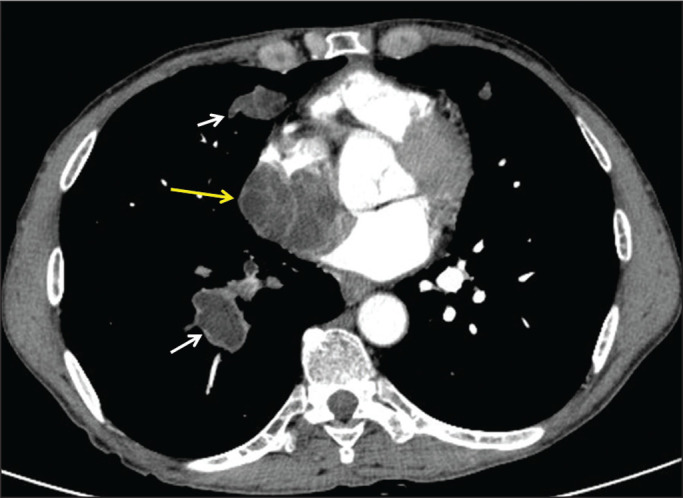
A 61-year-old male patient with a hydatid cyst resulting from contact with an animal. Chest CT showing a hydatid cyst with lobulated contours filling the right atrium (yellow arrow). Additional lesions consistent with hydatid cysts were detected in the lung (white arrows).

Among cases of cardiac hydatid cysts, only 3-4% involve the right atrium and only 6-8% involve the left atrium^(16)^. Cardiac hydatid cysts are generally asymptomatic; only 10% of patients develop symptoms^(16)^. In the case presented here, the patient had hydatid cysts in both lungs. A CT scan also revealed a hydatid cyst in the right atrium (Figure 8). The patient had not had any specific symptoms for atrial localization.

### Left ventricular involvement

**Figure 9:**
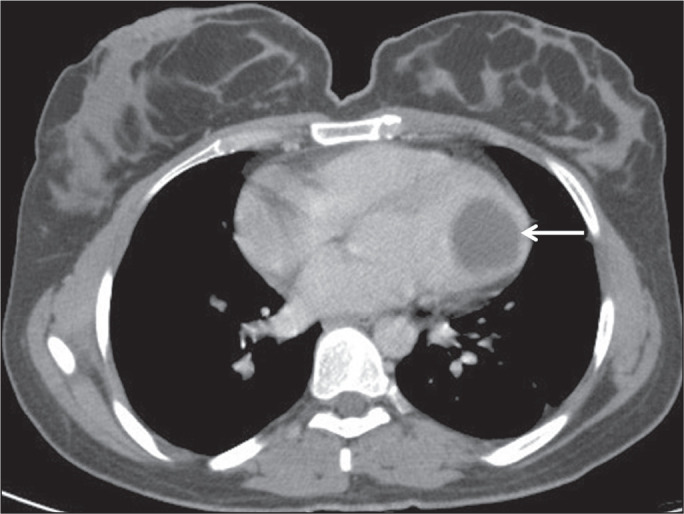
A 32-year-old female patient with a hydatid cyst of unknown origin. Contrast-enhanced CT of the chest, showing a lesion consistent with a hydatid cyst in the left ventricular apex (arrow).

Most cardiac hydatid cysts are located in the myocardium^(17)^, typically in the left ventricle (in 50-70% of cases), although they can also occur in the atrial walls (in 40-50%), the free wall of the right ventricle (in 30%), and the pericardium (in 15-25%). The most common symptoms are dyspnea, chest pain, and arrhythmias. Early diagnosis and treatment are important for preventing life-threatening complications. In the case presented here, a CT scan showed a hydatid cyst in the left ventricle (Figure 9).

### Involvement of the left ventricle and pericardium

**Figure 10:**
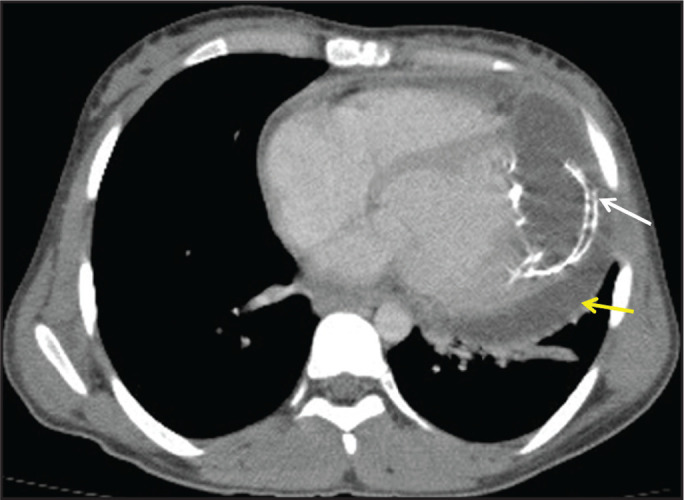
A 19-year-old female patient with a hydatid cyst of unknown origin. Contrast-enhanced CT of the chest, showing a calcified peripheral hydatid cyst with lobulated contours (white arrow) extending from the left ventricular apex toward the pericardial space. Effusion was also observed in the pericardial space (yellow arrow).

As mentioned above, 50-70% of cardiac hydatid cysts occur in the left ventricle and 15-25% involve the pericardium^(17)^. Patients with a hydatid cyst in the left ventricle, pericardium, or both typically present with arrhythmia and dyspnea. When a hydatid cyst is located in the pericardium, echocardiography indicates suspected neoplasia but does not provide sufficient information to make the differential diagnosis. In the case presented here, the patient had dyspnea and fatigue. Chest CT, performed in the cardiology clinic for the differential diagnosis of the mass, revealed a hydatid cyst in the left ventricle extending toward the pericardium (Figure 10).

## PARAESOPHAGEAL

**Figure 11:**
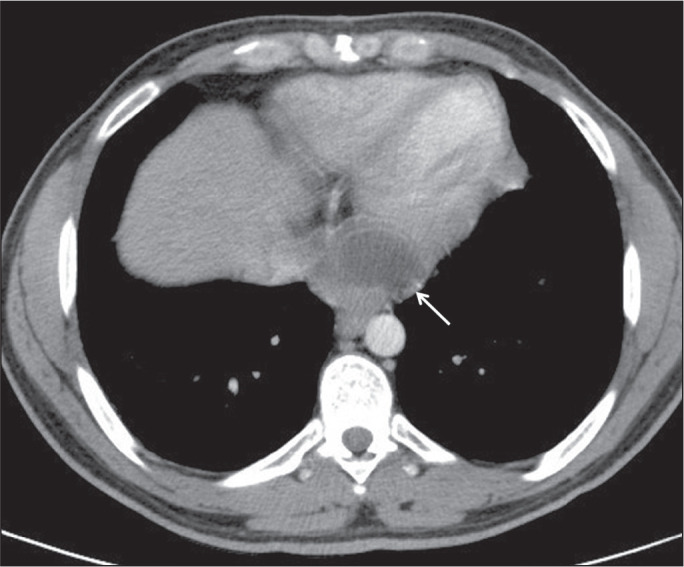
A 28-year-old male patient with a hydatid cyst resulting from contact with an animal. Contrast-enhanced CT of the chest, showing a lobulated lesion consistent with a hydatid cyst (arrow) in the anterior part of the esophagus.

Paraesophageal hydatid cysts are extremely rare. Patients with a paraesophageal hydatid cyst often present with the complaint of difficulty swallowing. In the case presented here, the patient presented with a six-month history of dysphagia. Chest CT showed a paraesophageal hydatid cyst (Figure 11), and the patient was admitted to the hospital. An enzyme-linked immunosorbent assay was positive for hydatid cyst fluid antigen, and the serum IgE level was high.

## CONCLUSION

Hydatid cyst should be considered in the differential diagnosis of a cystic lesion in any part of the body. Hydatid cysts have characteristic imaging findings that are essential for diagnosis. Therefore, radiological imaging may simply distinguish hydatid cysts from other infectious or neoplastic lesions. Although rare, hydatid cysts in atypical locations can provoke unusual complications, with unpredictable findings and symptoms.
